# Advancing Mental Health and Psychological Support for Health Care Workers Using Digital Technologies and Platforms

**DOI:** 10.2196/22075

**Published:** 2021-06-30

**Authors:** Jiancheng Ye

**Affiliations:** 1 Feinberg School of Medicine Northwestern University Chicago, IL United States

**Keywords:** mental health, health care workers, health informatics, digital intervention, health technology, mobile health, COVID-19

## Abstract

**Background:**

The COVID-19 pandemic is a global public health crisis that has not only endangered the lives of patients but also resulted in increased psychological issues among medical professionals, especially frontline health care workers. As the crisis caused by the pandemic shifts from acute to protracted, attention should be paid to the devastating impacts on health care workers’ mental health and social well-being. Digital technologies are being harnessed to support the responses to the pandemic, which provide opportunities to advance mental health and psychological support for health care workers.

**Objective:**

The aim of this study is to develop a framework to describe and organize the psychological and mental health issues that health care workers are facing during the COVID-19 pandemic. Based on the framework, this study also proposes interventions from digital health perspectives that health care workers can leverage during and after the pandemic.

**Methods:**

The psychological problems and mental health issues that health care workers have encountered during the COVID-19 pandemic were reviewed and analyzed based on the proposed MEET (Mental Health, Environment, Event, and Technology) framework, which also demonstrated the interactions among mental health, digital interventions, and social support.

**Results:**

Health care workers are facing increased risk of experiencing mental health issues due to the COVID-19 pandemic, including burnout, fear, worry, distress, pressure, anxiety, and depression. These negative emotional stressors may cause psychological problems for health care workers and affect their physical and mental health. Digital technologies and platforms are playing pivotal roles in mitigating psychological issues and providing effective support. The proposed framework enabled a better understanding of how to mitigate the psychological effects during the pandemic, recover from associated experiences, and provide comprehensive institutional and societal infrastructures for the well-being of health care workers.

**Conclusions:**

The COVID-19 pandemic presents unprecedented challenges due to its prolonged uncertainty, immediate threat to patient safety, and evolving professional demands. It is urgent to protect the mental health and strengthen the psychological resilience of health care workers. Given that the pandemic is expected to exist for a long time, caring for mental health has become a “new normal” that needs a strengthened multisector collaboration to facilitate support and reduce health disparities. The proposed MEET framework could provide structured guidelines for further studies on how technology interacts with mental and psychological health for different populations.

## Introduction

The COVID-19 pandemic, as a prolonged global public health crisis, has heavily burdened health care systems and the health care workers who are the direct responders to safeguard people’s health. Positive and optimistic emotional states play important roles in stimulating the human body's immune system, which could enable health care workers to effectively engage in the fight against the pandemic. Excessive pressure, anxiety, and depression can be detrimental to mental health and may prevent health care workers from actively performing their duties in response to the pandemic. The scale, pervasiveness, and complexity of the stressors associated with the ongoing pandemic are unprecedented, despite the fact that some countries have achieved milestones in controlling the pandemic and have moved forward to the initiation of vaccination. With the realization that the end of the pandemic is far from close, the toll of the pandemic on the mental health and well-being of health care workers still requires urgent attention.

Experiencing intense pressure at work for a long time may cause a series of problems that can affect physical and mental health, which can also affect workers’ quality of life and work efficiency [1,2]. The threat of being infected by the virus, inability to complete work, emotional impact of patients’ deaths, and concerns regarding the safety of family members all increase the emotional pressure on health care workers. Although vaccines have been distributed in some countries, research indicates that vaccine compliance remains variable and inconsistent [3,4]. The existing mental problems in the face of extensive media coverage of the rising numbers of casualties, overburdened health care systems, and psychological issues caused by the COVID-19 pandemic may have fostered health care workers’ anxieties and distrust in preventative health care. These fears could also result in vaccine hesitancy [3]. Given that the COVID-19 pandemic is expected to persist for a long time, caring for health care workers’ mental health has become a “new normal” that requires strengthened multisector collaboration to facilitate mental health support and reduce health disparities. However, to enhance their psychological preparedness for the new normal of the pandemic, there is a need to integrate resources and provide a more comprehensive and concerted psychological support for health care workers.

Understanding the risks of mental health issues that health care workers have been experiencing, identifying effective interventions to address the adverse effects of the pandemic, and proposing tailored strategies based on digital health will offer valuable support for health care workers. As we look to an uncertain future, a conceptual framework for the development and deployment of support will facilitate well-being endeavors and provide a foundation for addressing long-term mental health needs. The aim of this study is to develop a framework to describe and organize the mental health and psychological problems that health care workers are facing during the pandemic. Based on the framework, this study also proposes potential interventions from digital health perspectives that health care workers could leverage during and after the pandemic.

## Methods

### Conceptual Framework

**Figure 1:**
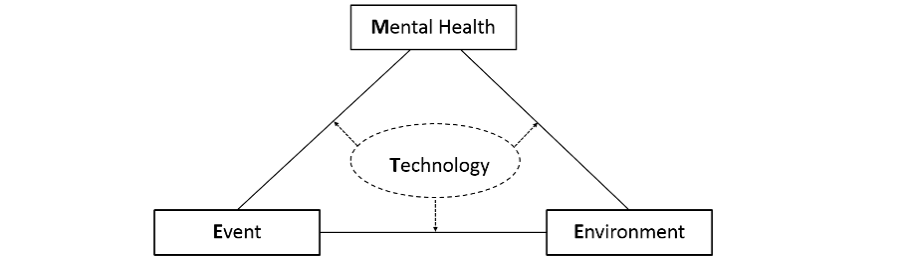
The MEET (Mental Health, Environment, Event, and Technology) framework.

In this study, we reviewed and analyzed the psychological problems and mental health issues that health care workers have encountered during the COVID-19 pandemic, and we developed the MEET (Mental Health, Environment, Event, and Technology) framework (Figure 1) to demonstrate the interactions of mental health, digital interventions, and social support.

There is a mismatch between the societal and organizational sources of psychological problems, such as lack of personal protective equipment, overwhelming workload, and the attempts by health care systems to address mental health issues at an individual level [5]. In this framework, mental health includes cognitive status, activities of daily living, behaviors, and instrumental activities of daily living. Environment refers to factors that are related to social support, family, and network composition. Events include the COVID-19 pandemic, lockdown, social distancing, and vaccine distribution [6]. Technology includes diverse types of digital interventions and platforms [7], such as online support forums, telehealth platforms, health apps, and wearable devices. Through the MEET framework, it is possible to better understand the interactions between mental health, event, environment, and technology.

### Search Strategy

The search strategy and selection criteria were designed to search the PubMed, Health Source: Nursing/Academic Edition, Embase database, and Scopus databases to identify relevant articles published up to the date of the study. Search terms included *COVID-19*, *2019-nCov*, *SARS-CoV-2*, *SARS-CoV*, and *coronavirus* and in combination with *health care worker*, *mental health*, *psychological health*, *technology*, and *digital intervention*. The search strategy also included Medical Subject Headings terms in the search strategy for PubMed and Emtree terms for Embase. The search was not restricted by study design. Textbox 1 demonstrates the search strategy based on the proposed MEET framework. This framework could also be applied to other populations by replacing section V with the corresponding population, such as older patients, pediatrics, etc.

**Search strategy outline based on the proposed MEET (Mental Health, Environment, Event, and Technology) framework.**

**I: Mental health**

1. Mental*

2. Psychol*

3. Psychiatric*

4. Anxiety

5. Depress*

6. Stress*

7. Psychosocial

8. Emotion*

**II: Environment**

1. COVID-19*

2. SARS-CoV-2*

3. Severe acute respiratory syndrome coronavirus 2

4. 2019-nCoV*

**III: Event**

1. Pandemic

2. Lockdown

3. Quarantine

4. Social distanc*

5. Infection

6. Vaccin*

**IV: Technology**

1. Technology

2. Digital intervention

3. Digital health

4. Digital platform

5. Informatic*

6. Health technology

**V: Population**

1. Health care worker*

2. Health care provider*

3. Health care professional*

Search strategy:

(I1 or I2 or I3 or I4 or I5 or I6 or I7 or I8) AND (II1 or II 2 or II 3 or II 4) AND (III1 or III2 or III3 or III4 or III5 or III6) AND (IV1 or IV2 or IV3 or IV4 or IV5 or IV6) AND (V1 or V2 or V3)

### Study Selection and Eligibility Criteria

This study included any type of study about any type of health care worker during the COVID-19 pandemic with outcomes relating to their mental and psychological health, as well as studies about digital health technologies and platforms. The prevalence of mental health issues and effects and the interventions aimed at preventing or reducing negative mental health issues were analyzed and summarized narratively. The search strategy imposed no restrictions on study design, methodology, or language. This study focused on the health care worker population and identified references by searching (title/abstract) using the keywords from the four domains (mental health, environment, event, and technology) that are listed in Textbox 1.

## Results

### Psychological Problems and Mental Health Issues of Health Care Workers

#### Anger

The inner sense of security of health care workers has been threatened by the global pandemic. Health care workers may feel helpless and powerless. From the psychological perspective, anger is a type of psychological defense [8]. There are multiple triggers of anger: anger with sudden outbreak, helplessness during the spread of the pandemic, delayed vaccination, etc.

#### Anxiety

Health care workers have received professional medical training, which can help them address the pandemic objectively and rationally. However, they also have the same emotional responses as the general population, who are experiencing feelings of anxiety and panic. In addition to worrying about themselves and their families being infected with SARS-CoV-2, some health care workers are worried that the pandemic will continue to spread. Some health care workers pay too much attention to negative news and information. When they feel physical discomfort, especially with respiratory symptoms, they often manifest anxiety, nervousness, and restlessness. This sense of losing control will likely result in overthinking, pessimism, loss of appetite, overeating, or weight loss [9].

#### Obsessive-Compulsive Habits, Traits, and Disorder

Obsessive-compulsive disorder [10] refers to a mental disorder with the main symptoms of repeated compulsive actions or forced thinking. In the current pandemic situation, hand washing, opening windows, and wearing masks are effective means to prevent SARS-CoV-2 infection. However, health care workers may engage in forced actions of excessive disinfection behaviors. They may overthink the negative consequences of the disease, while these abnormal behaviors can cause painful feelings.

#### Hypochondriasis

Hypochondriasis is a psychopathological status [11] in which a person ascertains that they have a specific disease without clear medical evidence. In the context of the COVID-19 pandemic, health care workers are in close proximity or direct contact with a large number of patients, and the potential risk factors for infection are significantly increased, which can lead to hypochondriasis. When physical discomfort occurs, the health care workers may overthink their symptoms, which may cause unnecessary anxiety and nervousness.

#### Depression

Pessimistic feelings are likely to trigger negative and hopeless emotions. These emotions are signs of depression. Various factors may contribute to depression, such as grief over the loss of lives, fear of becoming ill, and psychological trauma from the global pandemic [12].

#### Sleep Issues

Sufficient sleep is essential for health care workers to restore physical strength and improve immunity after high-intensity work. Having a good quality of sleep can reduce the risk of illness. However, during the pandemic, there are multiple barriers to sleep: isolation from society, disordered life rhythm, mental fatigue, depression, loss of interest and joy in life, etc. Health care workers may have difficulty falling asleep even when in an exhausted physical state, or they may experience shortened sleep time, frequent waking and dreaming, and disordered sleep rhythms.

#### Physical Discomfort and Somatization

During the pandemic, some health care workers may feel physical discomfort, which may be caused by physiological or mental health issues. Strong psychological fluctuation will lead to physical discomfort involving organ systems throughout the body. When health care workers are under great pressure, negative emotions tend to be transformed into physical symptoms, which is commonly called somatization [13]. With these symptoms, psychological disorders and pain may not be detected but may be present in the psychopathological process in the forms of physical discomfort or dysfunction. Common types of physical discomfort include palpitations, chest tightness, shortness of breath, airway obstruction, dizziness, bloating, fatigue, decreased appetite, unstable blood pressure, and menstrual disorders [14]. These experiences of physical discomfort tend to increase the tendency toward hypochondriasis and often lead to a sense of panic.

#### Cognitive Issues in Concentration

The human body will redistribute blood nutrients to the heart, muscles, and other organs when it is under stress. This process will reduce the essential supply to the brain and result in inattention, inability to focus, and decreased ability of judgment and perception. In addition, paranoia may be generated in such situations [15].

#### Behavioral Issues

In hospitals, when health care workers treat patients who are suspected SARS-CoV-2 carriers, and they are likely to be sensitive to patients’ coughing and prone to have conflicts with them. An irregular lifestyle, such as unhealthy diet, poor sleep, and lack of physical activity, will increase the likelihood of infection. Common behavioral problems include performance avoidance, decreased work enthusiasm and physical activity, increased dependence on families, and disorderly lifestyle and self-management. Health care workers may also experience unhealthy lifestyle activities, such as smoking, drinking, staying up late, and overeating; or blind behaviors, such as panic buying and stockpiling of disinfection supplies, food, drugs, etc.

### Mental Health and Psychological Protection Interventions

The COVID-19 pandemic has resulted in an increase in risk factors for mental health issues, which requires both short-term adaptations and sustained responses. Lack of training, social support, effective communication, and accommodative coping are common factors for developing psychological morbidities and adverse psychiatric outcomes [16]. Comprehensively integrated intervention approaches are often more effective than single treatment methods and have a longer-lasting effect. The emerging health information technologies [17] coupled with recent innovations in digital health could enable health services to offer tailored and proactive mental health care for health care workers.

#### Digital Communication Platforms

During the COVID-19 pandemic, health care workers are most likely to communicate with colleagues with whom they have been working together closely. This is because these colleagues are empathetic and understand the hardships and difficulties of frontline work, and their mutual consolation will be an effective intervention. Understanding and support from family are also important. However, due to the policy of social distancing, digital platforms may be more accessible during a pandemic [18]. These platforms could enable health care workers to communicate, which is an essential component of any universal, community-led response to the pandemic [19]. Furthermore, the digital communication platforms could provide a peer-support network for health care workers to share their emotional feelings, challenges, and personalized resolutions, which may foster resilience and comradeship.

#### Telehealth Platforms

Another ideal communication partner is a psychologist. Communicating with psychologists through telehealth or remote platforms can allow health care workers to express negative emotions, actively talk about the difficulties they face, and express personal feelings encountered during the work. Primary mental health care modes such as counseling, psychotherapy, or pharmacological treatment should be provided through the health care workers’ local health care system or organization as needed. Professional guidance from psychologists will help health care workers to relieve negative emotions, adjust their negative cognition, and restore a healthy mentality to enable them to better cope with work and interact harmoniously with their families. For those health care workers who are too busy to receive support from local psychologists, resources such as employee assistance programs, [20] crisis hotlines [21], and other institutional resources may be good first steps.

#### Self-Guided Psychological Interventions

Nonpharmacological interventions, such as cognitive-behavioral therapy, meditation, mindfulness, breathing, and relaxation training through websites or mobile apps, will be suitable for health care workers. Internet-based psychological intervention may be the most convenient, fast, and economical means for health care workers who are currently fighting the novel coronavirus. With the help of information technology, these interventions can be transformed into audiovisual interactions, which enables health care workers to access web-based psychological intervention without being restricted to a particular time and place. This media information can also transmit scientific psychological crisis response strategies to frontline health care workers effectively. In this way, health care workers can improve their mental health protection awareness and take timely action.

#### Internet-Based Interventions

Regularity, order, and a sense of control are effective means of coping with anxiety and panic. During the pandemic, despite the limited range and number of activities, health care workers are still expected to actively balance work and life. They should not overuse alcohol or tobacco to relieve pressure or negative emotions. Health care workers who have sleep issues need to pay attention to sleep hygiene and decrease their use of caffeine [22]. Studies have shown that evidence-based internet interventions can be helpful to address these issues [23]. For health care institutions that have not implemented internet-based interventions, providing mindfulness education or meditation interventions could significantly reduce stress and other psychological diseases [24,25].

#### Web-Based Learning Communities

Obtaining mental health knowledge through web-based learning communities is another effective approach. Emotions such as anxiety and fear are normal psychological reactions, and moderate anxiety can help people increase their awareness of prevention [26] and avoid dangerous environments. However, excessive pressure and anxiety will weaken the human body's immune system and damage its protection mechanisms. Receiving mental health education and training enables health care workers to make rapid and scientific judgments about their psychological status and offers them keen insights into abnormal psychological reactions.

This training includes education on the psychosocial impact of high-casualty events in different settings. Health care workers could develop a personalized resilience plan that involves the identification of anticipated responses. Meanwhile, they should also be taught how to use digital and mobile health technologies for delivering care [27. The earlier the intervention, the more likely that negative moods and psychological situations will be adjusted in time. Furthermore, this training will help health care workers understand stress-related obstacles and approaches to adjust their emotions in the face of catastrophic events as well as establish the correct psychological defense mechanism against crises. Although training and education may not generate an immediate effect, these efforts will create an active continuum of improved environment [28], reinforce the capacity to support increased access to care for mental health issues, and strengthen health care workers’ readiness for the new normal of the postpandemic era.

#### Artificial Intelligence in Health Care Systems

The COVID-19 pandemic has increased the stress of health care workers who were already overwhelmed by high workloads. Many health care workers are on the fringe of reaching their physical and psychological limits. High stress and overwork not only damage health care workers’ physical and mental health [29] but also affect their decision-making during clinical work [30]. Health care workers should objectively assess their own ability to withstand pressure and stress and measure their ability to devote themselves to effective work. Using artificial intelligence approaches, such as machine learning and deep learning [31], to plan a reasonable schedule of shifts and assist in clinical decision-making may help health care workers avoid physical and mental burnout [32].

#### Mobile Health

Point-of-care systems such as portable and smart devices [33], home diagnosis technologies based on the Internet of Things [34], and other digital interventions can help health care workers detect potential physical issues at early stages. In addition, these interventions could be tailored to health care workers and fit with their personal needs and lifestyles.

#### Short Videos

Health care workers are always seeking a transparent understanding of the situation during the pandemic [35]. Short videos provide a panoramic and detailed record of the actual situation. The intuitive ways in which they present information greatly improve the audience's acceptance and understanding [36]. Some short videos could provide advice on ways to stay healthy by teaching health care workers how to include sufficient physical activity in their routine, eat fresh food, and consume natural supplements that can support their immune systems. In addition, the short videos could facilitate wellness therapies to relieve stress, anxiety, and help health care workers maintain a general sense of mental and physical well-being.

#### New Media

The timely disclosure and dissemination of information could help health care workers and their families understand the course of the incident, the truth, and the real situation [37]. Meanwhile, authoritative information also eliminates rumors and prevents excessive pressure on health care workers. Higher satisfaction with disseminated public information may contribute to lower psychological distress. In the current situation, authoritative news could be quickly and widely disseminated through health communication technologies (ie, social media, short videos) to address public concerns. This information can strengthen the credibility of official departments and help reduce or even eliminate the influence of rumors [38]. New media platforms are also enhancing the affinity and attractiveness of digital approaches.

#### Social Media

Social media platforms are important sources for supervision of public opinion. During the pandemic, all departments, agencies, and institutions in society have been interlocked in their responses to the emergency, which requires an orderly, accurate, and efficient workflow. Mobile information and health communication technologies play prominent roles in media supervision, investigation, and filling in information gaps. Through mobile communication platforms, health care workers from different departments at the front line could share their perseverance, efforts, and strategies to prevent and control the pandemic situation from multiple perspectives [39].

Through social media, humanistic information and communication can not only calm health care workers and boost their confidence but also positively guide the public and help mitigate negative and anxious environments [40]. Social media is playing a comprehensive role in science popularization, as it is based on modern mobile communication technologies that are conveying scientific knowledge to the public in a fast, timely, and vivid fashion. With multiplatform and multichannel support to achieve rapid information coverage, the public can obtain a scientific understanding of the dynamic situation in a short time and mobilize their subjective initiatives for effective preventive actions, which is more efficient than passive installation [41].

## Discussion

### Principal Findings

Health care workers and professionals have the critical responsibilities of saving lives and protecting people's health during the COVID-19 pandemic. The pandemic has undoubtedly created universal psychological distress. Efforts to address the problem and to prevent the long-term mental health deterioration of health care workers are paramount in the response to COVID-19. Understanding the risks of mental health issues that health care workers are experiencing, identifying effective interventions to address the adverse effects of the pandemic, and proposing tailored strategies based on digital health will offer valuable support for health care workers. We provide a conceptual framework for allocation of the main sectors (mental health, environment, event, and technology) at the individual, organizational, and societal levels, focusing on addressing health care workers’ well-being needs during and after the pandemic.

To prepare for the long-term fight against the pandemic, these guardians of human life must maintain their physical and mental health to work effectively to take care of more patients. Providing health care workers with positive support will help mobilize their self-psychological protection capabilities, thus allowing them to continue their valuable work. The need for more mental health services will introduce additional burdens to health care systems, and digital health technologies are playing vital roles to relieve these overwhelmed systems. Leveraging hybrid solutions that offer web-based, telehealth-based, or blended face-to-face intervention and treatment may be more accessible and effective [42].

In addition to using digital technologies and platforms, health care workers should avoid information overload. Due to the modernization of communication approaches, the amount of information about the pandemic is overwhelming, which can increase the sense of insecurity and uncertainty. The traditional ways in which people obtain information, such as newspapers, radio, and television, have been transferred to the internet and mobile platforms such as social media, video, or live broadcast platforms. Mobile information and health communication technologies have revolutionized information dissemination, data exchange, media supervision, guidance of public opinion, and health communication [43]. Health care workers should pay more attention to authoritative information, actively avoiding negative news and preventing information from overwhelming them. Meanwhile, health care workers should also keep in regular contact with families and friends, which can not only increase emotional interaction and psychological support but can also increase mutual encouragement.

The pandemic may cause pressure, panic, and psychological trauma to health care workers. Technologies could not solve all the problems. Mild emotional distress can be adjusted by health care workers themselves, while serious panic will seriously impact their daily life. Self-regulation often has a limited effect and requires professional assistance, and it is not suitable for every health care worker, especially young health care providers who have not experienced such a serious crisis. Health care workers with insufficient clinical experience may generate more pressure and experience persistent depression, anxiety, insomnia, and other symptoms. Health care workers should request remote counseling from experts or go to a psychological clinic for consultation when necessary. If their psychological problems cannot be relieved after receiving professional psychological intervention or mental health services, psychiatrists should intervene in time and provide corresponding diagnosis and treatment. Given that the COVID-19 pandemic is expected to continue for a long time, caring for mental health has become a new normal that needs strengthened multisector collaboration to improve social support and reduce health disparities. To enhance the psychological preparedness of health care workers for the new normal of the pandemic, there is a need to integrate resources and provide them with more comprehensive and concerted psychological support.

### Conclusion

The COVID-19 pandemic has heavily burdened health care systems throughout the world. It is urgent and critical to protect the mental health and strengthen the psychological resilience of health care workers. The proposed MEET framework could aid understanding of the interactions among the mental health, event, environment, and technology sectors. In addition, this framework may provide structured guidelines for future research on mental and psychological studies for different populations. Long-term, proactive individual, organizational, and societal infrastructures to support health care workers’ mental health are needed to mitigate the psychological impact of the COVID-19 pandemic. Embedding these mental health practices as part of the new normal can be a stepping stone to a new future with benefits and implications for other global public health issues far beyond the response to the COVID-19 pandemic.

## Abbreviations

MEET: Mental Health, Environment, Event, and Technology
