# Correction: Advancing Mental Health and Psychological Support for Health Care Workers Using Digital Technologies and Platforms

**DOI:** 10.2196/84386

**Published:** 2025-10-08

Following the publication of “Advancing Mental Health and Psychological Support for Health Care Workers Using Digital Technologies and Platforms” [1], concerns were raised regarding corresponding author Jiancheng Ye’s rate of self-citation within the article’s reference list. Further investigation into this matter identified that numerous self-citations were added to the manuscript following acceptance of the article without adequate disclosure.

The author was contacted for a response regarding this matter. The author indicated that the added references were pertinent to the article and its findings, and provided documentation justifying the inclusion of the identified self-citations.

Following review of this response, the JMIR Publications Editorial Office determined that several of the added references were not relevant to the article or were used to support a generic statement where another reference could have been used instead of self-citation. Therefore, the JMIR Publications Editorial Office is issuing this corrigendum to remove the following references:

- Reference 1: Ye J. Health information system’s responses to COVID-19 pandemic in China: a national cross-sectional study. Appl Clin Inform 2021 Mar 19;12(2):399-406 [doi: 10.1055/s-0041-1728770] [Medline: 34010976]
- Reference 9: Ye J. Pediatric mental and behavioral health in the period of quarantine and social distancing with COVID-19. JMIR Pediatr Parent 2020 Jul 28;3(2):e19867 [doi: 10.2196/19867] [Medline: 32634105
- Reference 15: Ye J, Wang Z, Hai J. Social networking service, patient-generated health data, and population health informatics: patterns and implications for using digital technologies to support mental health. medRxiv. Preprint posted online June 18, 2021 URL: https://www.medrxiv.org/content/10.1101/2021.06.11.21258777v1
- Reference 19: Feinglass J, Wang JA, Ye J, Tessier R, Kim H. Hospital care for opioid use in Illinois, 2016-2019. J Behav Health Serv Res 2021 Jan 27:1-13 [doi: 10.1007/s11414-020-09748-8] [Medline: 33502670]
- Reference 32: Ye J, Ma Q. The effects and patterns among mobile health, social determinants, and physical activity: a nationally representative cross-sectional study. AMIA Jt Summits Transl Sci Proc 2021
- Reference 37: Ye J, Sanchez-Pinto LN. Three data-driven phenotypes of multiple organ dysfunction syndrome preserved from early childhood to middle adulthood. AMIA Annu Symp Proc 2020;2020:1345-1353 [Medline: 33936511]

The JMIR Publications Editorial Office regrets that these issues were not identified prior to publication.

The correction will appear in the online version of the paper on the JMIR Publications website together with the publication of this correction notice. Because this was made after submission to PubMed, PubMed Central, and other full-text repositories, the corrected article has also been resubmitted to those repositories.
